# Oxidative Profile of Patients with Sickle Cell Disease

**DOI:** 10.3390/medsci7020017

**Published:** 2019-01-25

**Authors:** Charles Antwi-Boasiako, Gifty B. Dankwah, Robert Aryee, Charles Hayfron-Benjamin, Eric S. Donkor, Andrew D. Campbell

**Affiliations:** 1 Department of Physiology, School of Biomedical and Allied Health Sciences, University of Ghana, Accra +233, Ghana; gdankwah@gmail.com (G.B.D.); Bobby200055@gmail.com (R.A.); chayfron-benjamin@ug.edu.gh (C.H.-B.); 2 Department of Anaesthesia, School of Medicine and Dentistry, University of Ghana, Accra +233, Ghana; 3 Department of Medical Microbiology, School of Biomedical and Allied Health Sciences, University of Ghana, Accra +233, Ghana; ericsdon@hotmail.com; 4 Center for Cancer and Blood Disorders Children’s National Medical Center George Washington University School of Medicine and Health Sciences, Washington, DC 20052, USA; acampbell@childrensnational.org

**Keywords:** sickle cell disease, catalase, superoxide dismutase, malondialdehyde

## Abstract

Oxidative stress plays a very significant role in the pathophysiology of sickle cell disease (SCD) and associated complications. Oxidative stress, which is often experienced by SCD patients as a result of continuous production of reactive oxygen species (ROS), may lead to endothelial dysfunction and acute inflammation. Antioxidant enzymes, such as superoxide dismutase (SOD) and catalase (CAT), often play a protective role. The current study aimed at determining the oxidative profile of persons with SCD at a tertiary hospital in Ghana. This was a case-control study involving 90 patients with SCD (34 HbSS patients at steady state, 30 HbSC at steady state, 15 HbSS with vaso-occlusive crisis, 11 HbSC with vaso-occlusive crisis), and 50 HbAA control group. Whole blood samples were collected from the study participants and analyzed for full blood counts. The blood samples were assayed for SOD and CAT as a measure of antioxidant defense, while lipid peroxidation was quantified as malondialdehyde (MDA). The results showed that the levels of SOD and CAT were significantly lower in SCD patients as compared to the control group. Patients with HbSS vaso-occlusive crisis had the lowest levels of SOD and CAT. The difference in SOD levels between HbSS at steady state and HbSC with vaso-occlusive crisis was, however, not significant (*p* = 0.228). The MDA level was significantly higher in SCD patients compared to the control group. This study concludes that the levels of various antioxidant enzymes (erythrocyte SOD and erythrocyte CAT) and oxidative marker (MDA) and are altered in SCD patients.

## 1. Introduction

Sickle cell disease (SCD) is an inherited blood disease with several complications in various populations worldwide. The disease is caused by a point mutation in the haemoglobin β (HBB) gene, which codes for β-globin, found on chromosome 11a. This mutation results in the substitution of the amino acid, valine for glutamic acid in the globulin protein, and leads to the production of an abnormal form of the haemoglobin molecule, which is the haemoglobin S (HbS) [1]. The disease afflicts about 20 million people worldwide, with a high prevalence of about 12 million people living in Africa [2]. The mortality rate for children under 5 years with SCD is estimated at 75–85% in Africa [3]. In Ghana, the prevalence of the disease is about 2% of births annually [4]. Vaso-occlusive crises (VOC) are a hallmark of SCD, which creates an economic burden and makes management of SCD patients difficult in developing countries [5]. The abnormal red blood cells, as a result of sickling, which is observed in SCD patients, may also lead to chronic intravascular haemolysis [6]. The free plasma haemoglobin released thus initiates peroxidation of lipids and further contributes to oxidative stress [7].

Oxidative stress plays a very significant role in the pathophysiology of SCD and associated complications [8,9,10,11,12]. Several mechanisms have been proposed to contribute to the increased oxidative burden in SCD patients. Some of the mechanisms include an excessive level of cell-free haemoglobin [13], chronic pro-inflammatory state [14], recurrent ischemia-reperfusion injury [8,11], higher auto-oxidation of HbS [15,16], and iron overload [17]. Previous studies have reported that higher levels of reactive oxygen species (ROS) are linked with the several SCD-related complications [10]. Besides, an increase in oxidative stress and an abnormal oxidant/antioxidant balance in the body have been reported to be a contributing factor in the pathophysiology of several complications in SCD patients [18,19,20], especially in patients with VOC [10]. Factors such as elevated intravascular haemolysis and chronic inflammation contribute to the production of increased ROS [13,14,15]. Although the body has its own mechanism of neutralizing increased ROS [21], the antioxidant defence may be overwhelmed by the large pool of ROS and may not effectively neutralize their effects in SCD patients. In Ghana, though several studies have investigated the pathophysiology of SCD, there is hardly any data related to oxidative stress. To address this research gap and contribute to the data on oxidative stress in SCD patients, this study was carried out. The aim of the current study was to determine the oxidative status of SCD patients at a tertiary hospital in Ghana.

## 2. Methods

### 2.1. Study Site, Design, and Sampling

This study was conducted at the Korle-Bu Teaching Hospital (KBTH) in the Greater Accra Region of Ghana. The KBTH is the largest hospital in Ghana with a bed capacity of about 2000 and 17 clinical and diagnostic departments, including a Sickle Cell Centre. This was a cross-sectional study involving 90 SCD patients and 50 controls from January to April 2018. The SCD subjects were recruited from patients who came for their routine hospital examination at the Sickle Cell unit of the KBTH. The controls were recruited from the National Blood Transfusion Centre located at KBTH. Recruitment of the study participants was based on clinical diagnosis of SCD; hemoglobin electrophoresis was used to confirm SCD phenotype and absence of sickle cell trait in controls. The clinical states considered in the present study were patients with vaso-occlusive crisis (VOC) as well as those in the steady state. Steady state was clinically defined as a patient who has been well and has not been in crisis for at least 2 weeks and is going about his or her activities. Vaso-occlusive crisis was clinically defined as a patient on admission at the sickle cell clinic for some hours, with pain in the bones, muscles, and joints not attributable to any other cause, and requiring parenteral analgesia. Vaso-occlusive crisis was diagnosed by the physician or haematologist on duty attending to the patients. Patients with obesity, metabolic syndrome, cancer, peripheral vascular disease (not only coronary), as well as autoimmune diseases, mainly lupus and rheumatoid arthritis, were excluded. Demographic characteristics and clinical history of the study subjects were extracted from their clinical history.

### 2.2. Laboratory Analysis

Venous blood (5 mL) was drawn with a 19G hypodermic needle fixed on a 5 mL syringe into vacutainer tubes and transported to the Human Physiology Laboratory of the Department of Physiology, College of Health Sciences, University of Ghana on ice. A full blood count was performed on all samples (*n* = 140) within 2 h of collection using Labsystems Multiskan MS (Amersham Bioscience Ltd., Little Chalfont, UK). The blood samples were processed into plasma, serum, blood cells, and buffy coat, and kept at −80 °C for analyses of the oxidative profile of the study participants. Erythrocytes were lysed with cold water and used for the assay. Malondialdehyde (MDA) was measured in serum while superoxide dismutase (SOD) and catalase (CAT) were measured in red blood cells (RBC). Lipid peroxidation was carried out by the method described by Ohkawa et al. [22] using the TBARS assay (R&D Systems, Minneapolis, MN, USA). In the presence of heat and acid, MDA reacts with thiobarbituric acid (TBA) to produce a coloured end product that absorbs light at 530–540 nm. The intensity of the colour at 532 nm corresponds to the level of lipid peroxidation in the sample. Levels of superoxide dismutase in the red blood cells were also determined using assay kits from Cayman chemicals, Ann Arbor, MI, USA. Superoxide dismutases are metallo-enzymes that catalyze the dismutation of the superoxide anion to molecular oxygen and hydrogen peroxide, and thus form a crucial part of the cellular antioxidant defence mechanism [23]. This kit utilizes a tetrazolium salt for the detection of superoxide radicals generated by xanthine oxidase and hypoxanthine. Levels of catalase in the red blood cells were determined using assay kits from Cayman Chemicals, Ann Arbor, MI, USA. Catalase is an enzyme present in lots of cells in the body, including the red blood cells. Catalase is involved in the detoxification of hydrogen peroxide to water and oxygen. This catalase assay kit utilizes the peroxidatic function of CAT for the determination of enzyme activity. The method is based on the reaction of the enzyme with methanol in the presence of an optimal concentration of H_2_O_2_. The formaldehyde produced is measured spectrophotometrically with 4-amino-3-hydrazino-5-mercapto-1,2,4-triazole (Purpald, Sigma-Aldrich, Sant Louis, MO, USA) as the chromogen [24].

### 2.3. Data Analysis

The data obtained from the study was entered into Microsoft Excel, 2010 and analyzed using SPSS version 20 software. Ordinal and nominal data were presented as frequencies. Results obtained were expressed as mean (±standard deviation), and *p*-values less than 0.05 were considered statistically significant. ANOVA was used to compare more than two means, followed by post hoc analysis where there was significance.

### 2.4. Ethics Statement

The study was conducted in accordance with the criteria set by the declaration of Helsinki and each subject signed an informed consent prior to participation. Ethical approval for the study was sought from the Ethical and Protocol Review Committee of the College of Health Sciences, University of Ghana (CHS-art/M.8-p3.2/2016-2017”, date 30 March 2017).

## 3. Results

### 3.1. Demographic Characteristics of the Study Participants

A total of 140 subjects were sampled. This was made up of 50 HbAA individuals (21 males, 29 females) with a mean age of 32.8 ± 10.4 years, 49 HbSS SCD patients (25 males, 24 females) with a mean age of 23.90 ± 8.04 years, and 41 HbSC SCD patients (18 males, 23 females) with a mean age of 34.17 ± 15.25 years. Of the 49 HbSS SCD patients that participated in the study, 34 were in the steady state whiles 15 had VOC. For the HbSC patients, 30 were in the study state while 11 had VOC.

### 3.2. Haematological Profile of the Study Participants

As shown in Table 1, the levels of haemoglobin (Hb) were higher in the control group. Patients with HbSS VOC had significantly lower levels of Hb. There was no significant difference in levels of Hb between patients with HbSC steady state and the control group (*p* = 0.958). The differences in levels of mean corpuscular volume (MCV), mean platelet volume (MPV), and platelet distribution width (PDW) among all the study groups were not significant. However, there were significant differences between the mean corpuscular hemoglobin (MCH) in the study groups (*p* = 0.0470).

### 3.3. Oxidative Stress Profile of the Study Participants

Table 2 shows the levels of oxidative stress markers of the study participants. The levels of SOD and CAT were significantly higher in the control group. Among the patients with SCD, the HbSS VOC group had lower levels of SOD and CAT. Patients with the HbSS genotype had lower levels of CAT and SOD compared with those with the HbSC genotype. The levels of SOD in HbSS steady state and HbSC VOC were not significantly different (*p* = 0.228). There were no significant differences in the levels of CAT between the control group (HbAA) and patients with HbSC steady state (*p* = 0.517), as well as those with HbSC VOC (*p* = 0.269). The difference in levels of CAT between HbSC steady state patients and those with HbSC VOC was also not significant (*p* = 0.910). The MDA level was significantly lower in the control group compared to the SCD group. It was observed that MDA level were higher in the HbSS VOC patients. MDA levels in steady states were significantly lower compared with those at the VOC state. A similar trend in the level of significance as in the case of CAT levels was observed in the MDA profile between the HbAA control group and HbSC steady state (*p* = 0.533), as well as those with HbSC VOC (*p* = 0.559).

## 4. Discussion

The generation of ROS contributes to the pathophysiology of SCD. These produced ROS result in oxidative stress and consequently lead to cellular damage of essential macromolecules, including DNA, proteins, and lipids [25]. The link between oxidative stress and disease complications has triggered the need for its study in haemoglobinopathies, including sickle cell. The mean serum MDA levels were significantly higher in HbSS patients as compared to HbSC and the HbAA controls. These findings are consistent with earlier studies, which reported higher serum MDA levels in SCD compared to controls [26,27]. The levels of oxidative damage measured as MDA was even more pronounced during VOC. This is because of the large amounts of ROS released during VOC, which causes even more pronounced oxidative damage. Reactive oxygen species destroy and alter lipid structure, leading to the formation of lipid peroxides, which were measured as MDA. The MDA levels therefore gives an idea of the extent of oxidative damage in the cells. The difference in levels of MDA in steady states and VOC patients can be explained partly by the degree of haemolysis in these patients, as well as the underlying chronic conditions. The difference in levels of MDA among patients with the HbSS genotype and those with the HbSC genotype partly suggest that the sickle cell genotype may affect the concentration of lipid peroxidation.

Activities of antioxidants, including SOD and CAT, help to combat the damaging effects of ROS [28]. This study reports that levels of SOD and CAT are significantly lower in patients with SCD as compared to controls. This is in line with other studies [27,29,30,31,32,33] that also reported levels of antioxidant enzymes to decrease in sickle cell patients. A previous study conducted by Manfredini et al. [34], however, reported a significantly higher level of SOD in the sickle cell anaemia (SCA) group compared to their healthy counterparts. In the same study, levels of CAT were also not significantly different between the SCA and controls. Nevertheless, previous studies have reported that oxidative stress is usually encountered in patients with SCA [35,36]. The lower levels of erythrocyte SOD and CAT may be due to the severity of oxidative stress in SCD subjects [27]. Haemoglobin S, which is present in the erythrocytes of SCD patients, auto-oxidizes faster as compared to normal haemoglobin and can result in the generation of more ROS, leading to more lipid peroxidation and may lead to the consumption or inactivation of antioxidant enzymes [37,38]. Superoxide dismutase and CAT are overwhelmed after ischemia and reperfusion, which may result in increased ROS in such patients [16]. Levels of antioxidant enzymes may, in part, depend on a combination of factors, such as the clinical state of patients, genotype, and the underlying chronic haemolysis. Results from the present study suggest that acute complications among SCD patients play a crucial role in their oxidative stress status. Patients with HbSS VOC had a significantly lower level of SOD, when compared to the other study subjects. The excessive amount of ROS produced during VOC may have partly contributed to the lower levels of SOD and CAT in these patients [29]. The difference in levels of SOD and CAT among HbSS steady and HbSC steady states, as well as those with HbSS VOC versus HbSC VOC, is partly due to the intensity of haemolysis, and the rapid auto-oxidation of the haemoglobin S molecule in those with the HbSS genotype [37,38]. Findings from the study also suggest in part that the lower levels of SOD and CAT in patients with the SS genotype may be due to significant depletion of antioxidants, such as nitric oxide and vitamins, in these patients. Lower levels of vitamin E [39,40] and nitric oxide [41] were reported in sickle cell patients with the SS genotype in previous studies. The non-significant differences in levels of SOD and CAT between some of the study participants is partly due to, perhaps, a similar ongoing haemolysis and associated conditions in the patients.

The haematological parameters herein assessed helped appreciate the haemolytic and chronic conditions experienced by the study participants. The trend in Hb, RBC, and HCT levels were expected due to the chronic haemolysis associated with SCD as well as a possible blood loss in haematuria. Our previous study reported that SCD patients with HbSS VOC have significantly lower levels of Hb and RBC counts compared to those in the steady state [42]. In this present study, we also noticed that of all the study participants, those with HbSS VOC had a significantly lower Hb level. The rate of RBC destruction is also very high in SCD and hence contributes to the lower levels of RBC and other RBC indices. It is not surprising to have observed that these HbSS VOC patients also had significantly lower SOD and CAT levels.

The main limitation of the study is the relatively small numbers of the different subgroups of SCD patients who were enrolled in the study, especially the HbSC VOC and HbSS VOC subgroup.

## 5. Conclusions

Sickle cell disease patients in this study recorded significantly higher levels of serum MDA compared to the controls, with HbSS patients in VOC recording the highest level of MDA. The erythrocyte CAT level was significantly lower in HbSS in the steady state and VOC as compared to controls. Although erythrocyte CAT levels were lower in HbSC as compared to controls, the difference was not significant. The study also revealed that mean erythrocyte SOD levels were significantly lower in SCD patients (both HbSS and HbSC in the steady state and VOC) compared with HbAA individuals.

## Figures and Tables

**Table 1:** Haematological parameters of study participants.

Parameters	Genotype of Subject					
	HbAA (*n* = 50)	HbSC Steady State (*n* = 30)	HbSC VOC (*n* = 11)	HbSS Steady State (*n* = 34)	HbSS VOC (*n* = 15)	*p*-Value
Hb	11.52 ± 1.50	11.30 ± 1.45	8.22 ± 0.77	8.94 ± 1.10	6.92 ± 0.63	<0.001 *
WBC	8.76 ± 3.39	6.45 ± 2.41	10.80 ± 5.46	9.36 ± 3.27	11.79 ± 5.01	<0.001 *
RBC	4.37 ± 0.57	4.47 ± 0.75	3.37 ± 0.60	3.32 ± 0.63	2.60 ± 0.47	<0.001 *
HCT	36.00 ± 4.49	36.11 ± 5.04	26.63 ± 2.41	28.25 ± 3.60	21.48 ± 2.27	<0.001 *
MCV	82.85 ± 6.89	81.27 ± 6.75	80.55 ± 10.71	86.11 ± 9.07	83.93 ± 10.59	0.1280
MCH	26.62 ± 2.50	25.52 ± 2.43	25.01 ± 4.27	27.56 ± 3.60	27.17 ± 4.29	0.0470 *
MCHC	32.00 ± 0.93	31.34 ± 0.91	30.88 ± 1.55	31.87 ± 1.06	32.24 ± 1.33	0.0020 *
RDW	14.94 ± 1.34	14.05 ± 1.42	15.18 ± 1.64	15.63 ± 1.84	17.90 ± 2.40	<0.001 *
PLT	296.29 ± 123.87	261.00 ± 91.56	384.18 ± 183.20	382.41 ± 100.80	464.60 ± 148.78	<0.001 *
MPV	7.87 ± 0.73	7.84 ± 0.81	7.82 ± 0.85	7.63 ± 0.70	7.65 ± 0.73	0.6110
PCT	0.23 ± 0.08	0.20 ± 0.06	0.30 ± 0.14	0.29 ± 0.08	0.35± 0.11	<0.001 *
PDW	12.63 ± 2.42	11.74 ± 3.36	11.62 ± 1.42	12.91 ± 2.03	12.09 ± 2.19	0.2710

Values are expressed as mean ± standard deviation (SD). Hb, haemoglobin; HCT, Haematocrit; RBC, red blood cell count; MCV, mean cell volume; MCH, mean cell haemoglobin; MCHC, mean cell haemoglobin concentration; RDW, Red cell distribution width; MPV, mean platelet volume; PLT, platelets count; PCT, plateletcrit; PDW, platelets distribution width; WBC, white blood cells. * significant at *p* ≤ 0.05.

**Table 2:** Levels of oxidative stress markers of the study participants.

Parameters	Genotype of Study Participants					
	HbAA (*n* = 50)	HbSC Steady State (*n* = 30)	HbSC VOC (*n* = 11)	HbSS Steady State (*n* = 34)	HbSS VOC (*n* = 15)	*p*-Value
**SOD**	7953.49 ± 595.46	7411.97 ± 384.53	6819.45 ± 380.85	6415.50 ± 525.50	5174.53 ± 825.73	<0.001 *
**CAT**	5.04 ± 1.97	4.49 ± 1.34	4.03 ± 1.10	2.45 ± 1.21	1.08 ± 0.66	<0.001 *
**MDA**	0.73 ± 0.68	0.99 ± 0.56	1.10 ± 0.48	1.29 ± 0.87	2.31 ± 1.06	<0.001 *

Values are expressed as mean ± standard deviation (SD); SOD, superoxide dismutase; CAT, catalase; MDA, Malondialdehyde. * significant at *p* ≤ 0.05.
